# Main factors determining the use of free MS-TRAM and DIEP flaps and comparing the results of breast reconstruction

**DOI:** 10.25122/jml-2022-0227

**Published:** 2023-01

**Authors:** Ivan Ivanovich Smolanka, Sergiy Petrovich Galych, Oleksii Volodimirovich Movchan, Irina Yuriivna Bagmut, Michael Ivanovich Sheremet, Igor Leonidovich Kolisnyk, Oleksandr Vasyliovych Bagmut, Andriy Oleksandrovich Lyashenko, Irina Viktorivna Dosenko, Oksana Mykolaivna Ivankova, Vitaliy Vasilyevich Maksymyuk, Volodimir Volodimirovich Tarabanchuk

**Affiliations:** 1 National Cancer Institute, Ministry of Health, Kyiv, Ukraine; 2 National Institute of Surgery and Transplantology, Kyiv, Ukraine; 3 Kharkiv Medical Academy of Postgraduate Education, Kharkiv, Ukraine; 4 Department of Surgery No.1, Bukovinian State Medical University, Chernivtsi, Ukraine; 5 Faculty of Computer Sciences, Karazin Kharkiv National University, Kharkiv, Ukraine

**Keywords:** autologous breast reconstruction, fat necrosis, MS-TRAM, DIEP-flap transplantations

## Abstract

This study aimed to compare the results of free MS-TRAM and DIEP-flap based on the volume of the transplant and the unique characteristics of blood flow in the tissues. The study included 83 patients, 42 in the MS-TRAM-flap reconstruction group and 41 in the DIEP-flap breast reconstruction group. In the MS-TRAM-flap group, 35 patients received delayed reconstruction, and 7 received one-stage breast reconstruction, including one case of bilateral transplantation. In the DIEP-flap group, 5 patients received one-stage reconstruction, and 36 received delayed reconstruction. Complications associated with the flap tissue were observed in 7 (16.67%) in the MS-TRAM-flap group and 8 (19.51%) cases in the DIEP-flap group. The total level of fat necrosis in MS-TRAM-flap was 7.14% (p=0.033), and in DIEP-flap, it was 9.75% (p=0.039) (2 patients had a substantial amount of fat necrosis, while 2 patients had a modest amount of focal fat necrosis). The number and diameter of perforators (including veins), as well as the transplant volume, are the primary determinants of whether to use a DIEP- or MS-TRAM-flap. DIEP-flap is preferred if there are 1–2 large artery perforators (≥1 mm) and tissue volume of 700–800 grams, while MS-TRAM-flap is used when the tissue volume is significant (>2/3 of standard TRAM-flap).

## INTRODUCTION

Breast cancer patients who undergo major surgery are typically recommended to have a mastectomy and one-stage or delayed reconstruction of the breast's shape and volume [1]. The effectiveness of this treatment depends on both overall survival and the standard of living. Various modified mastectomy techniques account for about 60% of all breast cancer procedures. The two most effective and used techniques for autologous breast reconstruction are free MS-TRAM-flap and DIEP-flap [2].

The main issue with these techniques is the risk of fat necrosis in the tissue, which is related to the idiosyncrasies of blood supply in various sections of the transplant. Ischemia of subcutaneous fat can lead to necrosis of fat cells and further scarring, which can simulate a relapse [3]. According to the literature, the risk of fat necrosis with TRAM-flap is 3.0%, and 42% when using tissue complexes from the lower abdomen wall (DIEP-flap) [4]. The total level of fat necrosis in MS-TRAM-flap was 7.14% (p=0.033).

Autologous breast reconstruction allows for a natural-looking breast with ductility, ptosis, and normal skin temperature, which is difficult to achieve with synthetic materials [5]. Lower front abdominal wall tissues are the most often used material for breast reconstruction (transverse rectus abdominis myocutaneous, or TRAM-flap) [6]. Free TRAM-flap transplantation can be done using three different modifications: the muscle-sparing or MS-TRAM flap, the deep inferior epigastric perforator or DIEP flap, and the myocutaneous TRAM flap. These transplants are comparable in terms of anatomical structure and tissue composition, allowing for the reconstruction of a breast with significant volume and good shape. They receive direct blood flow from the inferior epigastric artery [7].

According to the American Society of Plastic Surgeons, in 2015, DIEP-flap accounted for 42% of all autologous breasts [8]. The elevated risk of fat necrosis in DIEP-flap tissues, which can be as high as 35%, is attributed to several factors. One of the main factors is the reduced perfusion pressure in the transplanted tissue compared to TRAM-flap, caused by the use of one or more artery perforators. Additionally, the dissection of blood vessels connecting zones of blood flow (perforasomes) during flap harvesting can also contribute to the increased risk [9].

Indicators for problems in MS-TRAM- and DIEP-flap breast reconstruction, as well as their severity, have been well established. Clinical evidence demonstrates that both reconstruction techniques are trustworthy and safe and can maximize surgical outcomes when used appropriately [10].

The aim of this study was to evaluate the results of free MS-TRAM and DIEP-flap transplantations and to provide a description of the indications for each of these reconstructive techniques based on the volume of the transplant and the unique characteristics of blood flow in the tissues.

## MATERIAL AND METHODS

**Figure 1:**
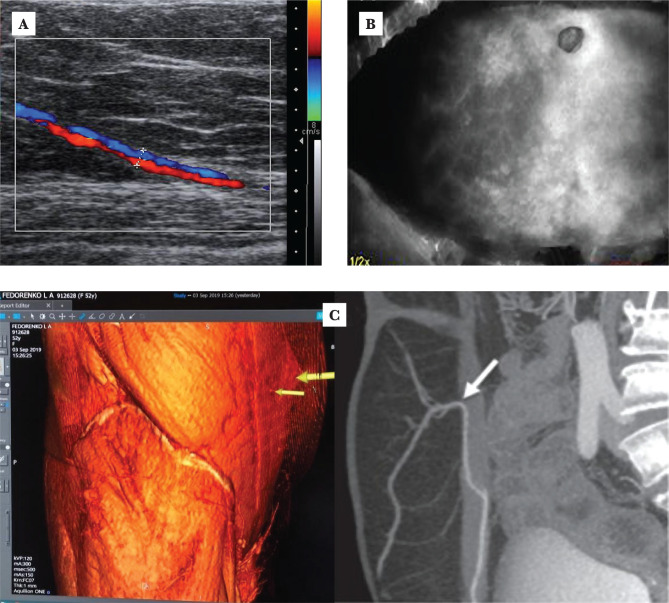
Preoperational diagnostics of location and parameters of major perforating vessels: A – ultrasonic duplex scanning; B – fluoptics fluobeam; C – computer tomography with angiography.

In this study, we evaluated the results of free MS-TRAM and DIEP-flap breast reconstruction in 83 patients following mastectomy, with ages ranging from 37 to 53 (median 45). 64 individuals underwent delayed repair, while 19 patients underwent one-stage surgery. All patients underwent standard clinical examinations in addition to having the angioarchitecture of the donor site and the condition of the arteries at the recipient site assessed. In order to achieve this, we used thermometry, computer and magnetic resonance imaging, and angiography. We were able to specify the parameters and preoperatively evaluate the state due to the analysis of the obtained data for well-done a. and v. mammaria internae, and a. and v. thoracodorsalis, and a. and v. epigastrica inferior, as well as the condition of the rectus abdominis and the degree of diastasis between them, and the position and characteristics of the most major artery perforators in the donor site (Figure 1 A–C).

**Figure 2:**
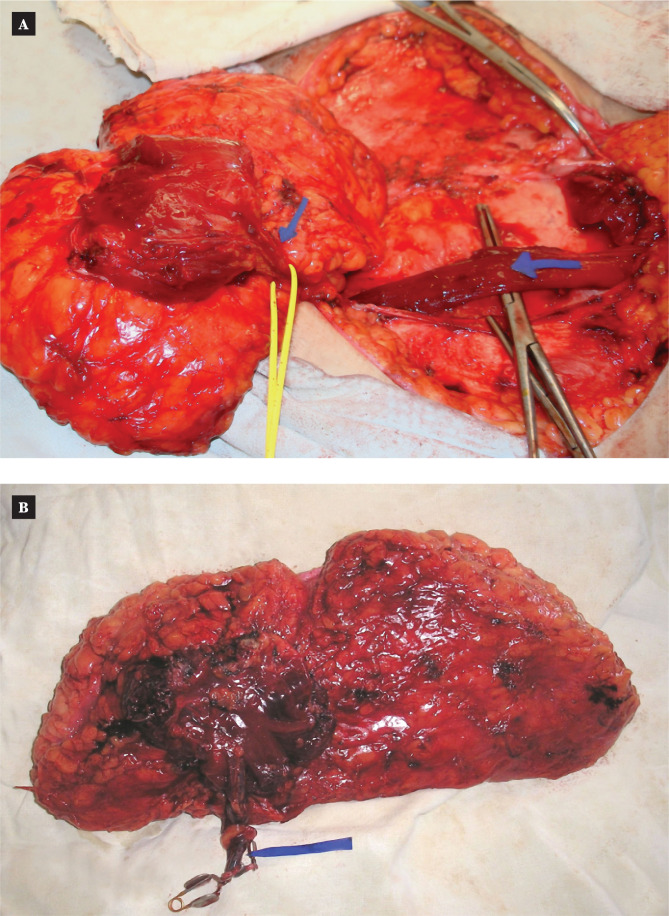
MS-TRAM-flap: A – flap harvesting on medial row of perforating arteries; B – tissue complex on two perforating arteries ready for transplantation.

The first group (MS-TRAM-flap reconstruction) consisted of 42 patients: 35 individuals received a delayed reconstruction, and 7 patients received a one-stage breast reconstruction (in one case, it was a bilateral transplantation). In some instances, the flap only contained a tiny piece of muscle and a portion of the anterior wall of the aponeurosis where the main artery perforators' outputs were located (Figure 2 A, B).

**Figure 3:**
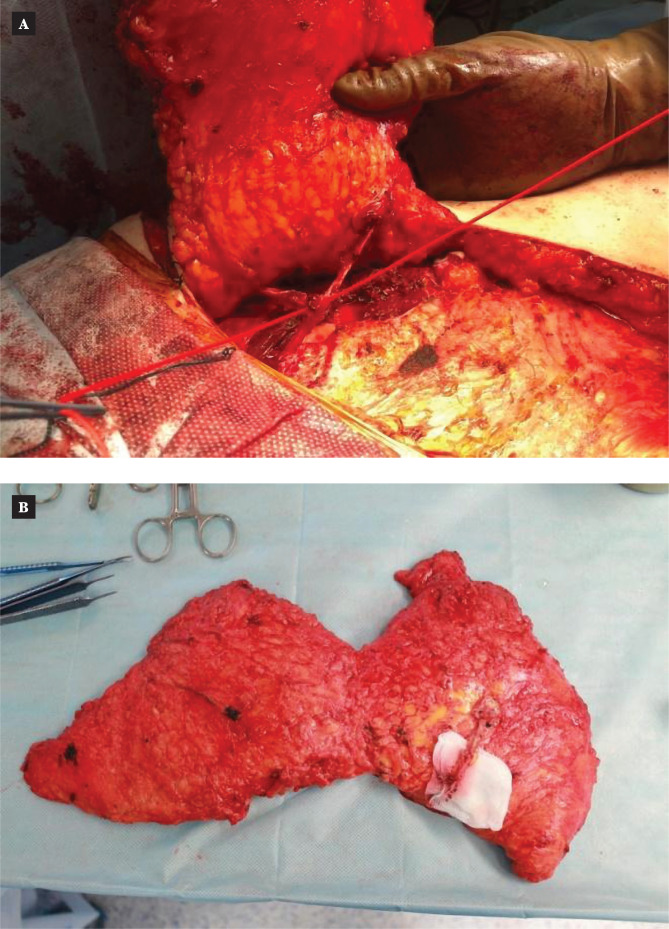
DIEP-flap: A –flap harvesting with one dominant perforating artery; B – tissue complex ready for transplantation.

The second group (DIEP-flap breast reconstruction) consisted of 41 patients, 5 of whom received one-stage breast reconstruction, and 36 received delayed surgery. In these cases, the flap did not include a muscular flap but had a thin muscular "muff" around the mobilized perforators (Figure 3 A, B).

We considered the amount of tissue removed after the mastectomy before deciding on a one-stage (immediate) reconstruction technique. The evaluation of initial characteristics, including the peculiarities of the tissue defect in the mastectomy zone, the condition of the surrounding tissue, the existence or absence of a sub-mammary fold, and the specifications of a contralateral breast, was crucial in a delayed (secondary) reconstruction. We also evaluated the state and volume of tissue in the donor site and the presence or absence of postoperative scars.

The "Technos MPX" (Escaote MyLab 25, Italy) with linear frequency sensors of 7, 5, 10, and 12, 5 MHz, as well as the "CE OPTIMACT 660", a high-speed multispiral computer tomograph that takes 64 section images for one turn of the radiator-sensor complex, were employed for the ultrasonic scanning. Indocyanine green (ICG) fluorescence imaging was created using Fluobeam fluoptics to visualize tissue perfusion. A thorough study of the received data was used to determine the best reconstruction technique.

For overweight and obese patients with strained abdominal muscles, a diastasis of more than 2 cm, and the need to reconstruct a breast of significant volume (>900 g), we selected MS-TRAM-flap [11]. Based on our prior clinical experience, DIEP-flap was primarily used for young patients with extra tissue in the anterior abdominal wall but without significant rectus abdomens muscle straining and a small or medium volume of a contralateral breast (up to 700–800 g). For nulliparous women, we categorically chose DIEP-flap because this transplant procedure created the least amount of disorder at the donor site and actually required an aesthetic abdominoplasty [12].

We employed the a. and v. thoraco-dorsalis as donor vessels in 6 cases of one-stage breast reconstruction and 3 cases of delayed breast reconstruction. In all other instances, *a. mammaria interna* was chosen because it provided a transplanted tissue with a higher perfusion pressure (26±12 ml/min) than a. thoraco-dorsalis (6±2 ml/min).

The nipple-areola complex was restored three to four months following the initial stage of reconstruction; if necessary, additional corrective procedures were carried out on the reconstructed breast (scar correction, sub-mammary fold, lipofilling) and healthy contralateral breast (augmentation and reduction mammoplasty, mastopexy).

To check on the health of the transplant tissue complexes, we used ultrasound control at the location of the micro anastomosis and generally accepted clinical assays. Standard control check-ups were performed in the delayed postoperative period every 1, 3, and 6 months.

Fat necrosis was recognized as a complication characterized by tissue indurations and nodules in the flap at least 1.5 cm in diameter and developed no sooner than 1–1.5 months after the procedure without affecting the surrounding dermis. Nahabedian M scale was used for a more objective evaluation of the fat necrosis degree: slight fat necrosis (<5% of volume), moderate (5–20%), and severe (>20%) [13].

Statistica version 7 was used to calculate medians, mean values 25^th^ to 75^th^ percentiles, and Spearman rank correlation coefficient (r). Additionally, multivariate Cox proportional hazard analyses only included statistically significant factors (p<0.05) in univariate analyses. Receiver operating characteristic (ROC) curve analysis was carried out using the software program IBM SPSS Statistics version 22.0.

## RESULTS

In the first group of MS-TRAM-flap breast reconstruction, complications associated with the flap tissue in the early postoperative period were observed in 7 (16.67%) patients. These included one case of total necrosis of the transplanted flap, 3 cases of partial necrosis, and 2 cases of marginal necrosis. In addition, three cases of assimilated flaps showed localized fat necrosis, with minimal necrosis in two cases and moderate necrosis in one. It is important to note that the transplant flaps of all 3 patients with fat necrosis contained some III zone tissue (Hazard Ratio (HR)=0.80, 95%, Confidence Interval (CI): 0.31–0.89, p=0.03) (Table 1).

**Table 1:** Complication frequency depends on the reconstruction variant.

Indicators	Venous congestion (n) (p<0.001)	Total necrosis of the transplanted flap (n) (p<0.008)	Partial and marginal necrosis (n) (p<0.014)	Fat necrosis (n) (p<0.001)
**MS-TRAM-flap**	0	1	5	3
**DIEP-flap**	3	1	3	4

It is necessary to point out that the number of artery perforators mobilized in MS-TRAM-flap in all cases was no less than 3 (with a diameter of up to 1 mm). Thus, the total level of fat necrosis in MS-TRAM-flap was 7.14% (p=0.033).

**Figure 4:**
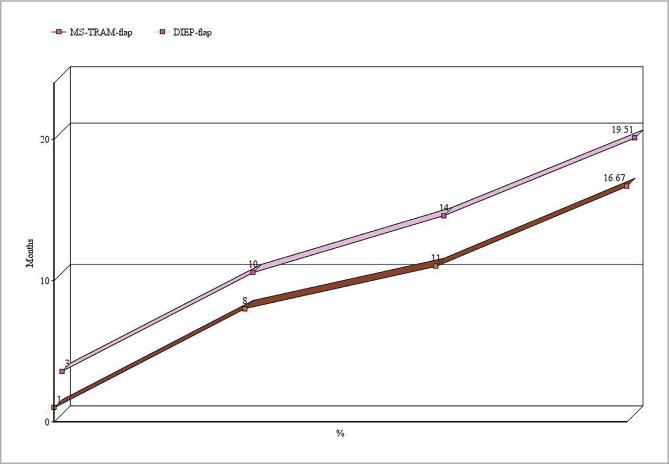
MS-TRAM-flap breast reconstruction complications compared to a group of DIEP-flap breast reconstruction complications.

In the second group of DIEP-flap reconstruction, 8 patients (19.51%) experienced different complications in the flap tissue during the early postoperative period. These included 1 case of total necrosis of the transplanted flap, 2 cases of partial necrosis, and 1 case of marginal necrosis. In the early postoperative period, 2 patients had a substantial amount of fat necrosis, while 2 patients had a modest amount of focal fat necrosis. Separate indurated nodules in the tissue at seams were ignored since they were linked to the body's response to absorbable threads, which was regularly noticed while carrying out other operations (HR=0.78, 95%, CI: 0.29–0.86, p=0.043) (Table 1). There were 3 cases of severe venous congestion reported after DIEP-flap inclusion in the tissue bloodstream, which required an additional imposition of anastomosis between v. epigastrica superficialis and superficial veins. It is important to note that 3 patients in the second group had only one large artery perforator (1.2, 1.5, and 2 mm). Overall the level of fat necrosis was 9.75% (p=0.039) (Figure 4).

## DISCUSSION

MS-TRAM-flap breast reconstruction complications were observed in 16.67% of patients (p=0.03) compared to 19.51% in the DIEP-flap group (p=0.043) (Figure 4). This corresponds to Dauplat J and colleagues [14], who found that patients over the age of 55 were more likely to experience early problems within the first eight weeks of recovery (p=0.022), particularly issues such as lymphocele/seroma (at the donor site: p=0.017, at the breast: p=0.049). The majority of these difficulties (10%) occurred within the first two months, with significant issues accounting for only 8% of reconstructions. The patient's age at surgery, personal history (diabetes, smoking, and overweight/obesity), prior relapse, adjuvant therapy, and the type of reconstruction were the main variables of our investigation, and Shakir *et al*. [15]. The average level of fat necrosis, according to 10,764 reconstructive procedures on 8,970 patients using tissue complexes from the lower abdomen wall, is 11.3%. The frequency of fat necrosis with TRAM-flap is 8.9%, while the frequency with DIEP-flap is 14.4% when instances with MS-TRAM-flap are removed [15].

The investigation of delayed surgical results, however, revealed that patients who underwent DIEP-flap breast reconstruction experienced less postoperative pain and fewer problems at the donor site resulting in a shorter hospital stay. This is consistent with the growing popularity of the DIEP-flap procedure in recent years, as noted by Martinez [16]; despite the DIEP flap being well known for having lower donor-site morbidity than other autologous methods, many patients need lengthy hospital stays, which drives up overall expenditures.

The results also revealed that there was no fat necrosis if the flap only included the tissue of the first and second zones, the transplant volume did not exceed 700 g, and there were 1 or 2 dominant (1.5 mm) artery perforators. However, the risk of fat necrosis increased with transplant volume: with a transplant volume of up to 900 g, there was fat necrosis with 3 arterial perforators in 3 cases, and with a flap volume of approximately 1000 g, there was fat necrosis with 4 perforators (1 diameter >1 mm and 3 – up to 1 mm) in 1 case. In other cases, fat necrosis developed in comparatively small transplants (up to 800 g) with 2–3 perforators, but artery perforators were 1 mm in diameter, corresponding to another study [17], where the standard group had 18 of 79 flaps (22.8 percent) with fat necrosis, whereas the indocyanine green group had only two of 58 flaps (3.4%; odds ratio, 0.11; 95 percent CI, 0.02 to 0.60; p=0.011). There were no other notable differences in the complication profile.

As a result of studying the complications rate, we found no statistically significant differences in the level and nature of complications in patients from both groups.

Our attention was drawn to DIEP-flap transplantation by signs of obstructed venous outflow, particularly in the flap's peripheral zone (17 observations). These symptoms usually appeared near the end of the procedure and went away 3–4 days later. The temporary spasm of thin committing veins during the harvesting of a perforating vascular bundle clearly explains it. However, as previously stated, due to venous congestion in three cases, we had to impose an additional anastomosis between v. epigastrica superficialis and a branch of a superficial vein in the recipient zone intraoperative. According to a number of authors, the outflow from tissues in the donor site comes primarily through the system v. epigastrica superficialis in such cases due to the peculiarities of the venous system structure, and minor vein perforators are unable to provide adequate venous drainage [18].

An important role appears to be played not only by mobilized artery perforators of sufficient diameter but also by tissue angioarchitectonic peculiarities and connections between separate "perforasomas". It is worth agreeing with the majority of researchers that if there is no dominant perforator (1.5 mm) and there are 4 perforators, MS-TRAM-flap is preferred [7].

In our study, we found no significant advantage of DIEP-flap over MS-TRAM-flap in assessing functional morbidity in the donor site. In our early research, we only found two cases of postnatal prolapse at the site of free TRAM-flap harvesting. It should be noted that in both cases, no synthetic implant was used; the rectus abdomens' muscle aponeurosis was primarily seamed. Following the use of small fragments of mesh, we found no functional deficiency of the abdominal wall. Other authors report no significant differences in the results of a "sit-up" test on patients with DIEP- or MS-TRAM-flap following a free TRAM- or MS-TRAM-flap harvesting. At the same time, some studies register frequent complications in the donor site with DIEP-flap harvesting, in particular, navel necrosis or long wound healing [19].

**Figure 5:**
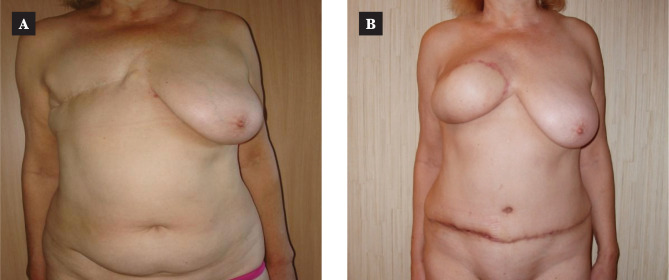
MS-TRAM-flap breast reconstruction: A – the patient before operation; B –the delayed result in 1 year.

**Figure 6:**
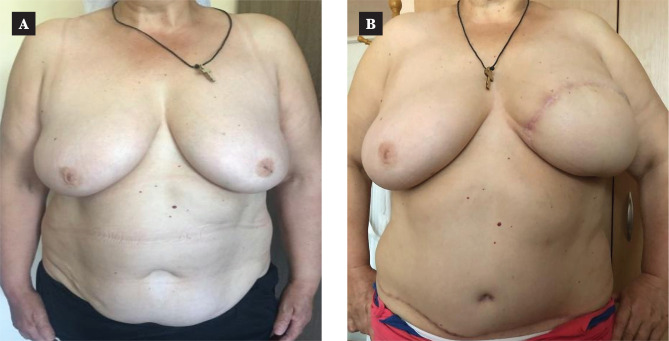
DIEP-flap breast reconstruction: A – the patient before the reconstruction; B –the delayed result in 2 years.

We did not find a reliable difference in evaluating this type of complication in the patients of both groups. The approach being reasonable and differentiated, both techniques provide a high-quality breast reconstruction (Figures 5 A, B and 6 A, B).

To achieve the best aesthetic results, oncologists and plastic surgeons must work together, and operations should be performed at specialized institutions with extensive experience in such interventions.

## CONCLUSIONS

The number and diameter of perforators (including veins), as well as the transplant volume, are the primary determinants of whether to use a DIEP- or MS-TRAM-flap. DIEP-flap is preferred if there are 1–2 large artery perforators (≥1 mm) and a tissue volume of 700–800 grams. MS-TRAM-flap is used when the tissue volume is significant (>2/3 of standard TRAM-flap), there is no dominant perforator, and 3–4 or more artery perforators are present. If the indicators are chosen correctly, both methods allow for maximum results
